# Antifungal susceptibility and virulence determinants profile of *candida* species isolated from patients with candidemia

**DOI:** 10.1038/s41598-024-61813-w

**Published:** 2024-05-21

**Authors:** Amal M. Dawoud, Sara A. Saied, Mohammad M. Torayah, Amira E. Ramadan, Shymaa A. Elaskary

**Affiliations:** 1 Department of Medical Microbiology and Immunology, Faculty of Medicine, Menoufia University, Shibīn El-Kom, Egypt; 2 Department of Clinical Pathology, National Liver Institute, Menoufia University, Shibīn El-Kom, Egypt; 3 Department of Anesthesia and Intensive Care, Critical Care Unit, Faculty of Medicine, Menoufia University, Shibīn El-Kom, Egypt; 4 Department of Medical Microbiology and Immunology, Faculty of Medicine, Benha University, Banha, Egypt

**Keywords:** Biofilm, *Candida* spp., Candidemia, Susceptibility, Virulence, Microbiology, Molecular biology

## Abstract

*Candida* is the most prevalent fungal bloodstream infection (BSI) with a high mortality rate among hospitalized patients. Another concern facing physicians is rising global incidence of drug-resistant *Candida.* This study aimed to characterize the prevalence, antifungal susceptibility, biofilm formation, and virulence genes (HWP1, ALS1, SAP2) of different *Candida* spp. isolated from patients with candidemia. 52 isolates of *Candida* spp. were identified from blood cultures by chromogenic *Candida* agar and confirmed by the VITEK 2 system. Isolates were tested for antifungal susceptibility by disk diffusion and VITEK 2 system. Biofilm formation and investigated genes were detected by the Congo red method and conventional PCR, respectively. *Candida* spp. caused 2.3% of detected BSIs, of which 32.7% were caused by *Candida albicans* (*C. albicans*) and 67.3% by non-*albicans Candida* (NAC), with the predominance of *C. tropicalis* (25%), followed by *C. parapsilosis* (17.3%), and *C. krusei* (13.5%). The susceptibility rates to fluconazole, voriconazole, caspofungin, micafungin, amphotericin B, and flucytosine were 64.7%, 76.5%, 100.0%, 100%, 100.0%, and 100.0% in *C. albicans,* while 53.6%, 71.4%, 91.4%, 91.4%, 94.3%, and 94.3% in NAC, respectively. Biofilm production, HWP1, ALS1, and SAP2 were detected in 70.6%, 82.4%, 76.5%, and 52.9% of *C. albicans* and 74.3%, 85.7%, 80.0%, and 48.6% of NAC, respectively. There is remarkable shift to NAC BSIs and high azole resistance. Antifungal stewardship and analysis of risk factors associated with this shift are needed.

## Introduction

Invasive fungal infections in immunocompromised individuals have been commonly linked to *candida* bloodstream infections (BSIs) in hospital settings. *Candida* is the fourth most prevalent cause of nosocomial BSIs in the US, the third detected cause of fungal sepsis in Europe and had a 37% mortality rate within 30-day duration^1^.

An increased risk of candidemia in immunocompromised individuals has been linked to a number of variables, such as broad-spectrum antibiotics, chemotherapy, neutropenia and invasive interventions. Blood cultures are the most reliable method for detection of candidemia. Nevertheless, physicians need to base their diagnosis on clinical picture and the existence of risk factors due to false negative results, long time required for diagnosis and the potential harmful effects of delayed or ineffective antifungal therapy^2^.

Despite the fact that *C. albicans* is still regarded as a significant pathogen of candidemia, an ongoing shift from *C. albicans* to non-*albicans Candida* (NAC) spp. was reported by several countries. *C. albicans, C. tropicalis, C. parapsilosis, C. krusei and C. glabrata* are responsible for over 90% of cases of candidemia. More unusual species including *C. guilliermondii, C. lusitaniae, and C. kefyr* have recently been known to induce candidemia, which poses a new risk to hospitalized patients' health^3^.

*Candida* infections often get therapeutic failure, mostly as a result of antifungal resistance that is caused by several mechanisms including biofilm production as biofilm-producing strains show significant increased resistance to antifungal drugs and host immunity^4^.

Numerous genes are reported to promote biofilm formation including hyphal wall protein1 (*HWP1*), agglutinin-like sequence (*ALS1*) and *ALS3 *genes. Also, release of extracellular hydrolytic enzymes make *candida* more virulent. For example, the release of aspartic proteinases (Saps), a family of ten enzymes, promotes cell wall proteins cleavage that facilitates the penetration of deeper epithelial layers and adhesion to host tissues. Furthermore, fungus release of phospholipases in order to break down phospholipids, a significant part of the cell membrane, may help in host tissue invasion. The expression of the *SAP1, SAP2, SAP3, SAP4, SAP5, SAP6, PLB1*, *PLB2,* and *LIP1-10* genes controls the synthesis of these enzymes^5^.

Patients’ underlying medical conditions have an impact on *Candida* spp. distribution and their antifungal susceptibility profile, which differs geographically. Consequently, choosing an empirical antifungal therapy for candidemia requires an understanding of the local pathogenic spectrum and changes in susceptibility^6^.

Our aim was to detect prevalence, antifungal susceptibility, biofilm formation and virulence genes (*HWP1, ALS1* and *SAP2*) of different *Candida* spp. isolated from patients with candidemia.

## Methodology

### Study design, setting and subjects

This cross-sectional study was conducted in the Faculty of Medicine and National liver institute, Menoufia University during the period from June 2022 to January 2024 and included patients from different Departments (ICU, oncology, dialysis, pediatric and transplantation unit). Patients were subjected to full history taking and clinical examination. Each patient provided written informed consent and the study was performed according to the Declaration of Helsinki. The study protocol has been approved by the Ethical Committee, Faculty of Medicine, Menoufia University (ANET 14-2). Demographic and clinical data including primary illness and risk factors at the time when blood culture was positive were all collected.

### Isolation of *Candida*

Blood culture bottles which alarm positive were subcultured on Sabouraud dextrose agar (SDA) medium supplemented with chloramphenicol (0.5 g/l) (Oxoid, UK) then incubated for 24–48 h at 37 °C. Colonies were then identified by standard methods (colony morphology, Gram stain and germ tube)^7^. Identifying *Candida* spp. in at least one positive blood culture from patients having symptoms and signs of infection was considered BSI with *Candida*^8^. A patient with multiple episodes of candidemia had one specimen included. Blood cultures from patients having incomplete records were excluded. Nutrient broth supplemented with 20% glycerol was used to preserve confirmed *Candida* isolates at – 80 °C^9^.

### Identification of *Candida* spp.

According to manufacturer guidance, HiCrome™ *Candida* Differential Agar (Himedia, India) was used to facilitate provisional rapid differentiation of *candida* spp. within 48 h of incubation at 37 °C based on colony morphology and color. VITEK 2 compact System (bioMerieux, France) was then used to confirm the results using identification cards YST.

### Antifungal susceptibility testing

Following the Clinical and Laboratory Standards Institute^10^, disk diffusion method was used to assess the antifungal susceptibility of *candida* isolates then confirmed by VITEK 2 system using sensitivity cards AST-Y08. The utilized antifungal disks (LIOFILCHEM, Italy) included fluconazole (FLU) 100 µg, voriconazole (VO) 1 µg and caspofungin (CAS, 5 μg). The reference strains *C. albicans* ATCC 90028, was employed as quality control.

### Detection of biofilm formation with Congo red agar method

*Candida* isolates producing black colonies on Congo red agar during a 48-h incubation at 37 °C, were considered biofilm forming isolate^11^.

### Genotypic detection of virulence genes (*HWP1*, *ALS1* and *SAP2*) by conventional multiplex PCR

Using the QIAamp DNA Mini Kit 50 tests (Qiagen, Germany, cat. no. 56304), *Candida* DNA was extracted and purified as per manufacturer’s instructions. With the use of a Nanodrop ND-1000 spectrophotometer (NanoDrop Technologies, USA), the amount and quality of the DNA were examined. The sequences of used primers and PCR conditions were followed as previously described by^12,13^ (Table 1). The PCR amplification was done using pre-programmed thermal cycler (Biometra, Germany). Electrophoresis for 20 min was done using agarose gel 2% (EGY technology) that was stained with the dye ethidium bromide (Sigma, USA). The gene products were seen using a UV trans-illuminator and 100 bp DNA ladder (Cleaver scientific, UK).

**Table 1:** Primers and PCR conditions used for PCR for detection of investigated genes.

Target Gene	Primer Sequence (5′-3′)	PCR conditions	Size (bp)	Reference
*HWP1*	**F:** CCATGTGATGATTACCCACA	Initial denaturation: 1 cycle of 94 °C for 4 min, Denaturation: 35 cycles of 94 °C for 30 s, Annealing: 52 °C for 1 min, Extension: 72 °C for 2 min. A final extension cycle was performed at 72 °C for 5 min	572	Inci et al.
	**R:** GCTGGAACAGAAGATTCAGG			
*ALS1*	**F:** CCATCACTGAAGATATCACCACA		318	
	**R:** TGGAGCTTCTGTAGGACTGGTT			
*SAP 2*	**F:** AACAACAACCCACTAGACATCACC		178	Lima et al.
	**R:** TGACCATTAGTAACTGGGAATGCTTTAGGA			

### Ethical approval

This experiment was approved by the National Liver Disease Institute's Research Ethics Committee (ANET 14-2). The Declaration of Helsinki's essential principles and practices were followed throughout the research.

### Consent to participate

Every participant gave their written authorization after being informed of the study's objectives and any potential negative side effects.

## Results:

**Figure 1:**
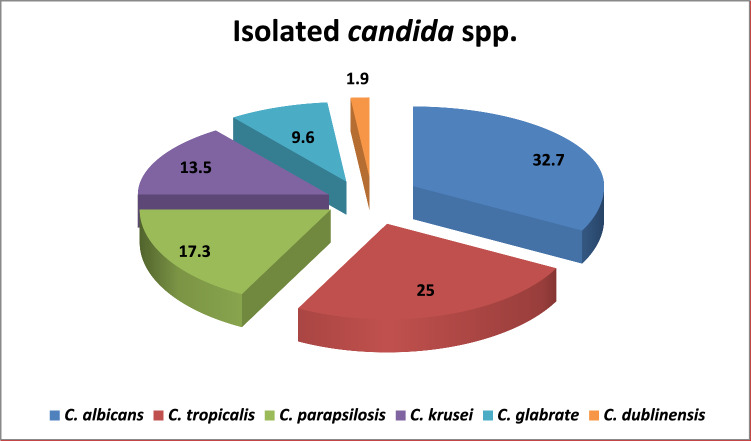
Distribution of isolated *candida* spp*.* among patients with *candidemia.*

**Figure 2:**
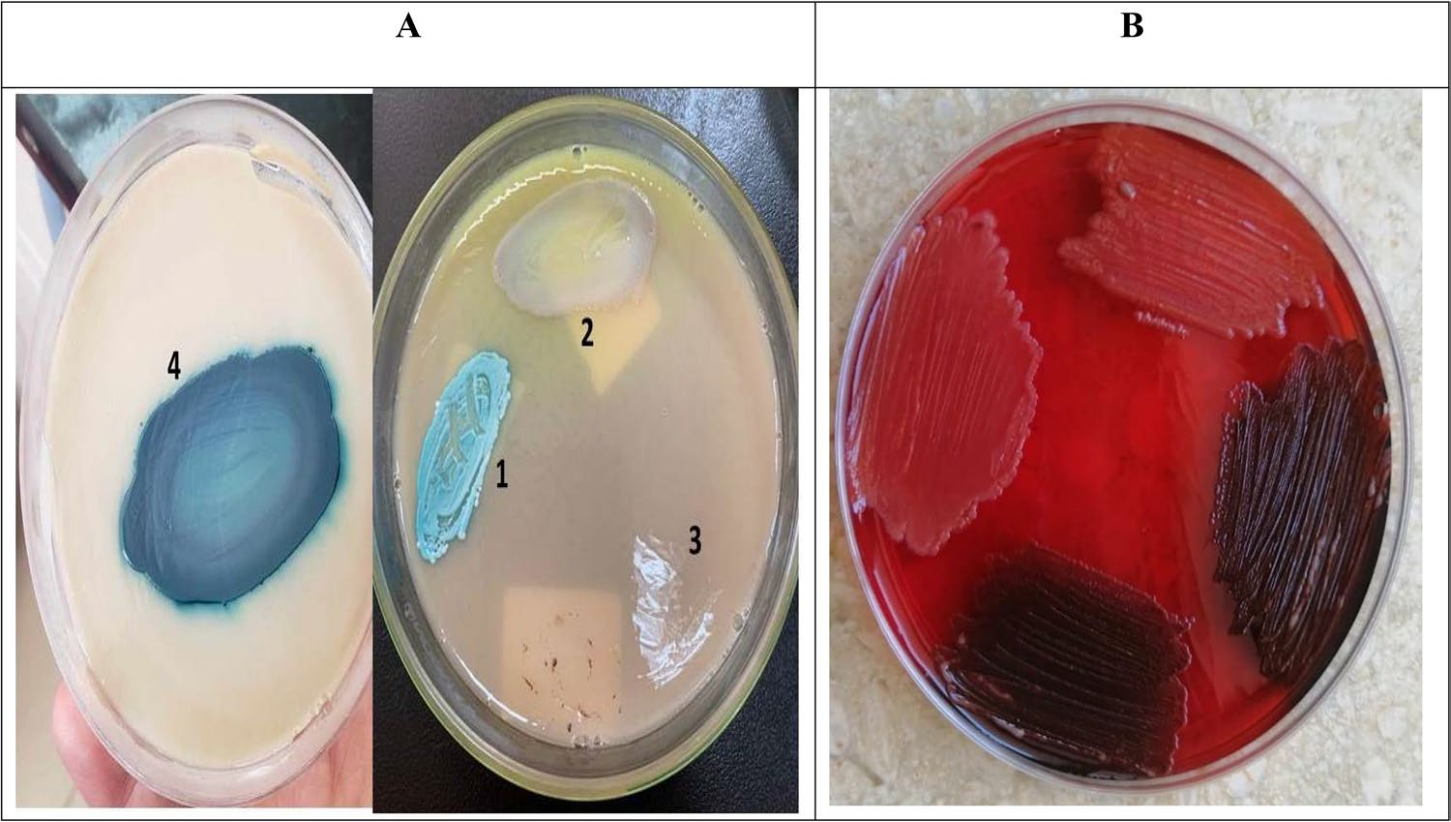
(**A**) Identification of *candida* spp*.* by Chromogenic *Candida* agar. 1, *C. albicans*; 2,* C. krusei*; 3, *C. parapsilosis*; 4,* C. tropicalis.* (**B**) Detection of biofilm production by congo red agar method*:* black colonies for biofilm producing isolates while red colonies for non-biofilm producing isolates.

Of all detected BSIs, 2.3% (n = 52/2272) were caused by *candida. C. albicans* represented 32.7% (*n* = 17/52) while NAC constituted the majority 67.3% (*n* = 35/52) of isolated *candida* spp. with the predominance of *C. tropicalis* 25% (*n* = 13/52) followed by *C. parapsilosis* 17.3% (*n* = 9/52) and *C. krusei* 13.5% (*n* = 7/52) (Figs. 1 and 2A).

Among our patients, the potential risk factors for candidemia included antibiotic therapy (82.7%), invasive procedure exposure (75.0%), prolonged hospital stay (> 7 days) and malignancy (69%). Sex, risk factors and ward of isolation didn’t significantly (P value > 0.05) affect distribution of *C. albicans* and NAC. However, most of isolated NAC was from elderly (57.1%) and very young patients (22.9%) while most of isolated *C. albicans* (70.6%) was from patients aged 10–60 years old with highly statistically significant difference (P value = 0.001). most of isolated *Candida* spp. were from ICU (38.5%) and oncology unit (28.8%) (Table 2).

**Table 2:** Demographic and clinical characteristics of patients with *candidemia.*

	Total (n = 52)	*C. albicans* (n = 17)		Non-*albicans candida* (n = 35)		χ^2^	P value
		NO.	%	NO.	%		
Age: mean ± SD	44.21 ± 25.20	36.11 ± 21.45		48.14 ± 26.22		13.73	0.001 HS
< 10	11 (21.1)	3	17.6	8	22.9		
10–60	19 (36.5)	12	70.6	7	20.0		
> 60	22 (42.3)	2	11.8	20	57.1		
Sex							0.61 NS
Male	28 (53.8)	10	58.8	18	51.4	0.25	
Female	24 (46.2)	7	41.2	17	48.6		
Risk factors							
Antibiotic treatment	43 (82.7)	12	70.6	31	88.6	2.58	0.10 NS
Invasive procedure	39 (75.0)	10	58.8	29	82.9	3.52	0.06 NS
Malignancy	36 (69.2)	9	52.9	27	77.1	3.14	0.07 NS
Leucopenia	16 (30.8)	4	23.5	12	34.3	0.62	0.43 NS
Prolonged hospital stay (> 7 days)	39 (75.0)	11	64.7	28	80.0	1.42	0.23 NS
Ward							0.63 NS
ICU	20 (38.5)	8	47.1	12	34.3	2.54	
Oncology unit	15 (28.8)	3	17.6	12	34.3		
Pediatric	11 (21.2)	3	17.6	8	22.9		
Dialysis unit	4 (7.7)	2	11.8	2	5.7		
Transplant unit	2 (3.8)	1	5.9	1	2.8		

**Figure 3:**
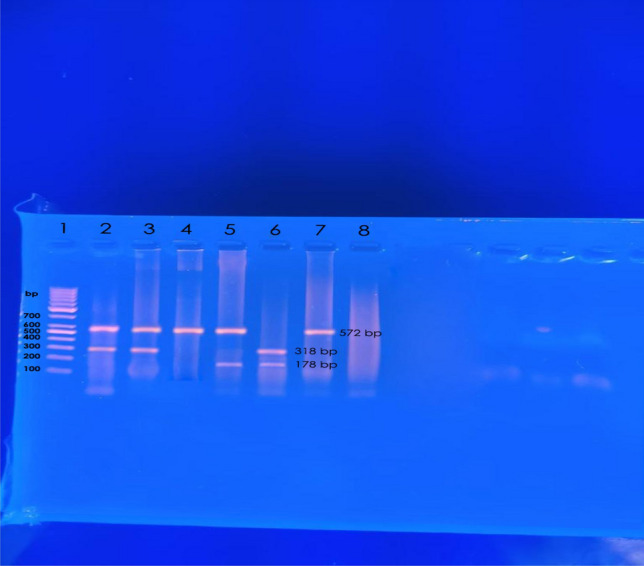
Gel electrophoresis showing the amplified product of the *HWP1* (572 bp)*, ASL1* (318 bp)* and SAP2* (178 bp) genes. Lane 1: 100 bp DNA ladder (Cleaver Scientific, UK). Lane 2, 3, 4, 5 and 7: *HWP1* positive isolate; Lanes 2, 3 and 6: *ALS1* positive isolates; Lane 5 and 6: *SAP2* positive isolates; Lane 8: negative control.

*C. albicans* showed higher susceptibility to antifungal drugs and lower biofilm formation compared to NAC but with no statistically significant difference (P value > 0.05). Susceptibility rate to fluconazole**,** voriconazole**,** caspofungin**,** micafungin**,** amphotericin B and flucytosine was *64.7%, 76.5%, 100.0%, 100%, 100.0% and 100.0% in C. albicans* while 53.6%, 71.4%, 91.4%, 91.4%, 94.3% and 94.3%% in NAC respectively. *C. glabrata* was more frequently resistant to the azole antifungals. The majority of *C. albicans* (70.6%) and NAC (74.3%) were biofilm producers. *HWP1, ALS1* and *SAP2* were detected in 82.4%, 76.5% and 52.9% of *C. albicans* and 85.7%, 80.0% and 48.6% of NAC respectively (Table 3, Figs. 2B and 3).

**Table 3:** Antifungal susceptibility and virulence determinants of *candida* isolates*.*

	*C. albicans *(n = 17)	Total NAC (n = 35)	*NAC*					χ^2^*	P value
			*C. tropicalis* *(n* = *13)*	*C. parapsilosis* *(n* = *9)*	*C. kruzei* *(n* = *7)*	*C. glabrata* *(n* = *5)*	*C. dublinensis* (n = 1)		
Antifungal susceptibility rate									
Fluconazole	*11 (64.7%)*	15 (53.6%)	*8 (61.5%)*	*5 (55.6%)*	*---*	*2 (40.0%)*	*0 (0.0%)*	0.53	0.46 NS
Voriconazole	*13 (76.5%)*	25 (71.4%)	*10 (76.9%)*	*7 (77.8%)*	*5 (71.4%)*	*3 (60.0%)*	*0 (0.0%)*	0.14	0.70 NS
Caspofungin	*17 (100.0%)*	32 (91.4%)	*12 (92.3%)*	*9 (100.0%)*	*7 (100.0%)*	*4 (80.0%)*	*0 (0.0%)*	1.54	0.21 NS
Micafungin	*17 (100.0%)*	32 (91.4%)	*12 (92.3%)*	*9 (100.0%)*	*7 (100.0%)*	*4(80.0%)*	*0 (0.0%)*	1.54	0.21 NS
Amphotericin B	*17 (100.0%)*	33 (94.3%)	*13 (100.0%)*	*9 (100.0%)*	*6 (85.7%)*	*5(100.0%)*	*0 (0.0%)*	1.01	0.31 NS
Flucytosine	*17 (100.0%)*	33 (94.3%)	*13 (100.0%)*	*9 (100.0%)*	*6 (85.7%)*	*5(100.0%)*	*0(0.0%)*	1.01	0.31 NS
MIC 50 (Range)									
Fluconazole	≤ 2.0 (0.25–32)		≤ 1.0 (0.5–32)	≤ 2.0 (0.25–16)	NA	≤ 64.0 (8.0–128.0)	NA		
Voriconazole	≤ 0.062 (0.031–4.0)		≤ 0.062 (0.015–4.0)	≤ 0.062 (0.031–2.0)	≤ 0.25 (0.12–8.0)	≤ 16.0 (0.12–32.0)	NA		
Caspofungin	≤ 0.12 (0.031–0.25)		≤ 0.12 (0.062–4.0)	≤ 1.0 (0.5–2.0)	≤ 0.25 (0.062–0.25)	≤ 0.12 (0.062–2.0)	NA		
Micafungin	≤ 0.12 (0.015–0.25)		≤ 0.062 (0.031–2.0)	≤ 0.50 (0.25–1.0)	≤ 0.12 (0.015–0.25)	≤ 0.062 (0.031–1.0)			
Amphotericin B	≤ 0.12 (0.031–0.5)		≤ 0.12 (0.031–0.5)	≤ 0.25 (0.12–0.5)	≤ 0.12 (0.015–4.0)	≤ 0.12 (0.062–64.0)	NA		
Flucytosine	≤ 0.12 (0.015–0.5)		≤ 0.12 (0.007–0.25)	≤ 0.12 (0.062–0.25)	≤ 0.25 (0.031–64.0)	≤ 0.25 (0.031–0.5)	NA		
Biofilm production rate	12 (70.6%)	26 (74.3)	12 (92.3%)	8 (88.9%)	5 (71.4%)	0 (0.0%)	1 (100.0%)	0.07	0.77 NS
Virulence genes									
*HWP1*	*14 (82.4%)*	30 (85.7%)	*13 (100.0%)*	8 (88.9%)	*6 (85.7%)*	*2 (40.0%)*	1 (100.0%)	0.09	0.75 NS
*ALS1*	*13 (76.5%)*	28 (80.0%)	*13 (100.0%)*	*9 (100.0%)*	*5 (71.4%)*	*0 (0.0%)*	1 (100.0%)	0.08	0.77 NS
*SAP2*	*9 (52.9%)*	17 (48.6%)	*9 (69.2%)*	*4 (44.4%)*	*3 (42.9%)*	*0 (0.0%)*	*1 (100.0%)*	0.08	0.77 NS

Significant values are in [italics].

*Comparison between *C. albicans* and Total NAC.

*---C. kruzei* has intrinsic resistance to fluconazole.

**Table 4:** Association between investigated genes and biofilm formation among *candida* isolates*.*

	*C. albicans *(n = 17)		Non-*albicans candida* (n = 35)		χ^2^	P value
	Biofilm forming (n = 12)	Non-Biofilm forming (n = 5)	Biofilm forming (n = 26)	Non-Biofilm forming (n = 9)		
Virulence genes						
* HWP1*	12 (100.0)	2 (40.0)	25 (96.2)	5 (55.6)	8.74^a^ 8.99^b^	0.003^a^ 0.002^b^
* ALS1*	11 (91.7)	2 (40.0)	24 (92.3)	4 (44.4)	5.23^a^ 9.57^b^	0.02^a^ 0.001^b^
* SAP2*	8 (66.7)	1 (20.0)	15 (57.7)	2 (22.2)	3.08^a^ 3.36^b^	0.07^a^ 0.06^b^

^a^Comparison of biofilm and non-biofilm forming isolates of* C. albicans.*

^b^Comparison of biofilm and non-biofilm forming isolates of Non*-albicans candida.*

*HWP1* and* ALS1* genes were significantly (P value < 0.05) higher among biofilm forming *C. albicans* and NAC isolates compared to non-biofilm producing isolates. However, *SAP2* gene was higher among biofilm forming *C. albicans* and NAC isolates but with no statistically significant difference (P value > 0.05) (Table 4).

## Discussion

In hospital settings, candidemia continues to be the most common invasive fungal infection with a high rate of morbidity and mortality. Studies that directly compare the epidemiology and treatment approaches of different countries are rare in real life and have the potential to provide more focused insights on enhancing clinical outcomes^14^.

In the current study, 2.3% of BSIs was caused by *candida* spp. This is nearly matched with previous studies of Alkharashi et al.^8^ in* KSA* (2.8%) and El-Mahallawy et al.^6^ in Egypt (3.1%). Higher prevalence of candidemia were reported among pediatric patients by studies presented by Karaağaç et al.^15^ in Turkey (12.9%), Khairat et al.^16^ in Egypt (17.3%). An Egyptian study performed by Reda et al.^3^ documented that 1.6% of BSIs in adults and 10.8% in children were caused by *Candida* spp.

The exact species distribution among patient with candidemia display considerable geographical, hospital-to-hospital, and even unit-to-unit diversity because of risk factors and practices. Despite *C. albicans* is still the most frequently isolated species, a progressive shift to NAC spp. is recently reported in most parts of the world^17^. Furthermore, species distribution might be affected by the kinds of antifungal drugs empirically used. Fluconazole use has been shown to increase the risk of *C. glabrata* and *C. krusei* infections, whereas caspofungin increases the risk of *C. parapsilosis, C. glabrata,* and* C. krusei* infections^18^.

In agreement with our results, previous studies in different countries reported *C. albicans* to be the primary cause of candidemia^8,15,16,19–21^. However, higher rates of *C. albicans* isolation among patients with candidemia were detected in other studies in KSA (51.3%)^22^, Turkey (39.42%)^23^ and USA (67%)^24^. In accordance, two different Italian studies in a large Italian University and tertiary hospitals found that 43.63% and 61.2% of candidemia caused by *C. albicans* respectively^25,26^. In contrast, El-Mahallawy et al.^6^, Muderris et al.^27^ and Treviño-Rangel et al.^28^ found that *C. tropicalis* (36.7%), *C. parapsilosis* (49.1%) and* C. tropicalis* (52.8%) respectively were the predominant species followed by *C. albicans*.

Regarding distribution of NAC in this study, there is predominance of *C. tropicalis* 25% followed by *C. parapsilosis* 17.3% and *C. krusei* 13.5%. This is in line with previous studies^3,29,30^. In contrast, in studies in Israel and Nordic countries *C. glabrata* was the principal NAC causing candidemia^31,32^. In a study conducted in Egypt, *C. krusei* was the most predominant NAC (28%) followed by *C. parapsilosis* (20%) and *C. tropicalis* (16%) among ICU patients ^33^. In several studies *C parapsilosis* was the most frequent NAC in children^26,34,35^.

In the present study, age had significant effect on *Candida* spp. distribution among patients. In previous studies analyzing species distribution in association with patient age, they observed that *C. glabrata* was the most prevalent isolate in elderly patients, while *C. parapsilosis* was more frequent in younger patients. *C. albicans* and *C. tropicalis* were isolated from all age groups^36,37^.

Yardimci et al.^24^ reported that micafungin sensitivity was the highest (97.4%) while fluconazole showed lowest sensitivity (66.1%) in 236 isolates which is consistent with this study. Similarly, an Egyptian study^6^, reported that resistance to fluconazole and voriconazole was 58.3% and 16.7% respectively among *Candida* BSIs. Fluconazole resistance in NAC and *C. albicans* were 64.3% and 50.0% respectively. This agreed with another Egyptian study conducted at Cairo University on pediatric patients in which they found high resistance rate to fluconazole among *C. albicans* (38.9%) and NAC (44.0%) causing BSIs^16^. Another study in ICU at Ain Shams University Hospital, Egypt, showed that all identified *candida* spp., including *C. albicans*, *C. krusei*, *C. glabrata*, *C. tropicalis*, and *C. parapsilosis* had high resistance to voriconazole and fluconazole (38.4% and 38.5%; 21.5% and 100.0%; 100.0% and 40.0%; 12.5% and 25.0%; and 10.0% and 20.0%, respectively^33^. In Solomon et al., study ^38^, all *Candida* isolates were susceptible to caspofungin, micafungin, amphotericin B and voriconazole. While *C. parapsilosis* and *C. auris* showed complete resistance to fluconazole and *C. tropicalis* was resistant to flucytosine.

Our data revealed that the majority of isolates (> 70%) were biofilm producers and positive for biofilm related genes *(HWP1* and* ALS1*). In agreement, Brunetti et al.^23^ stated that 69.28% of *Candida* BSIs were biofilm producers with the highest intrinsic production among *C*. *albicans* and *C*. *tropicalis*. Additionally, Treviño-Rangel et al.^28^ discovered that all the strains (89 isolates) produced biofilm with the highest biofilm density among *C. tropicalis*.

Further studies with a larger number of isolates, preferably multicenter studies, should be performed to confirm our findings and retrieve more statistically significant results. Also further researches were needed to discuss the association of *Candida* spp. virulence determinants (biofilm capacity and their related genes) with the clinical course and prognosis of affected patients. Additionally, relation between prophylaxis with antifungal drugs and the prevalence of *Candida* spp. should be investigated in future researches.

## Conclusion

There is remarkable rising incidence of NAC BSIs and high rates of fluconazole resistance highlighting the need for continuous candidemia surveillance, antifungal stewardship to maintain antifungal efficacy and analysis of risk factors associated with shift to NAC candidemia. Future research into the molecular mechanisms underlying azole resistance is recommended.

## Data Availability

The datasets present in the current investigation can be upon a reasonable request.
